# ﻿Four new species of *Diglyphomorphomyia* Girault (Hymenoptera, Eulophidae) from China, with a key to Chinese species

**DOI:** 10.3897/zookeys.1095.80671

**Published:** 2022-04-13

**Authors:** Jun-Jie Fan, Cheng-De Li

**Affiliations:** 1 School of Forestry, Northeast Forestry University, Harbin, 150040, China Northeast Forestry University Harbin China

**Keywords:** Chalcidoidea, Eulophinae, parasitoid, taxonomy

## Abstract

Four new species of *Diglyphomorphomyia* Girault, *D.depressa***sp. nov.**, *D.fossa***sp. nov.**, *D.hainana***sp. nov.**, and *D.octoseta***sp. nov.**, are described from China. A key to the eight species of the genus *Diglyphomorphomyia* occurring in China is provided.

## ﻿Introduction

The genus *Diglyphomorphomyia* (Hymenoptera: Eulophidae) is one of several small genera of the Eulophidae, erected by Girault (1913) with *Diglyphomorphomyianigriscutellum* Girault as type species. Girault (1913, 1915) also described *Sympiesomorphelleusalbiclava* Girault and *S.specimenipennis* Girault from Australia, which were later transferred by Bouček (1988) to *Diglyphomorphomyia*, together with *Chiloneurusrufescens* Motschulsky. Zhu and Huang (2003) described four species from China; Ubaidillah (2003) described one species from Indonesia; Yefremova (2007) described one species from Yemen; Narendran (2004, 2011) and Narendran et al. (2005a, b) described eight species from India. Currently the genus contains 18 valid species (Noyes 2021).

This study describes four new species of the genus *Diglyphomorphomyia* and provides a key to all species of the genus distributed in China.

## ﻿Materials and methods

All specimens were collected by sweeping or yellow-pan trapping, and were dissected and mounted in Canada balsam on slides following the method of Noyes (1982), or mounted on a card. Photographs were taken with a digital CCD camera attached to an Olympus BX51 compound microscope or Aosvi AO-HK830-5870T digital microscope. Measurements were made using an eyepiece reticle, or using the ruler tool in Adobe Photoshop 2020.

Terminology follows the Hymenoptera Anatomy Consortium (2021), and the following abbreviations are used:

**F1–6** flagellomeres 1–6;

**MV** marginal vein;

**OOL** minimum distance between a posterior ocellus and corresponding eye margin;

**PMV** postmarginal vein;

**POL** minimum distance between posterior ocelli;

**SMV** submarginal vein;

**STV** stigmal vein.

All type material is deposited in the insect collections at Northeast Forestry University (**NEFU**), Harbin, China.

## ﻿Results

### ﻿ Diglyphomorphomyia

Taxon classificationAnimaliaHymenopteraEulophidae

Girault

E01ED123-4FD7-5799-A720-F7CD4364F817

#### Diagnosis.

*Diglyphomorphomyia* can be recognized by the following combination of characteristics: funicle 4 or 5 segmented; midlobe of mesoscutum or scutellum with deep punctures or pits; midlobe of mesoscutum with two or three, rarely four, pairs of setae; scutellum with sublateral grooves very distinct, punctate on bottom; propodeum with anterior margin raised into a perpendicular lamina, median carina and plicae distinct; transverse carina lateral to plicae distinct behind propodeal spiracle; gaster subsessile or short-petiolate.

#### Description.

Head in frontal view a little wider than its height; Antenna with six flagellomeres, including a 2-segmented clava; antenna inserted above level of lower eye margin, scape not reaching level of anterior ocellus. Eyes with short setae. Malar sulcus present. Pronotum without transverse carina, weakly reticulate, posterior margin with numerous setae and six long setae. Scutellum punctate. Dorsellum with engraved reticulation, meshes isodiametric. Propodeum smooth, with median carina and plicae, a transverse carina running from posterior part of each plica to outer margins of propodeum. Gaster subsessile.

### ﻿Key to Chinese species of *Diglyphomorphomyia* based on females

**Table:** 

1	Notauli strongly divergent posteriorly meeting inner angle of axillae; dorsellum smooth (Fig. 11 page 447 of Zhu and Huang 2003)	***D.metanotalia* Zhu & Huang**
–	Notauli straight, converging posteriorly and meeting scuto-scutellar suture laterad to inner angle of axillae; dorsellum reticulate (e.g., Figs 1, 5)	**2**
2	Propodeum with median carina bifurcate anteriorly; midlobe of mesoscutum with 4 pairs of long setae (Fig. 17)	***D.octoseta* sp. nov.**
–	Propodeum with median carina not bifurcate anteriorly (e.g., Figs 1, 5); midlobe of mesoscutum with 2 or 3 pairs of long setae	**3**
3	Scutellum with a median groove or a fovea anteriorly (e.g., Figs 1, 5)	**4**
–	Scutellum without median groove or fovea (e.g., Fig. 15)	**5**
4	Antenna with scape, pedicel and F1 yellow and remainder of flagellomeres dark brown (Figs 6, 10)	***D.fossa* sp. nov.**
–	Antenna yellowish with F3 and F4 brownish, club brownish except yellowish apex (Fig. 2)	***D.depressa* sp. nov.**
5	Body dark brown to black	***D.nigra* Zhu & Huang**
–	Body mostly yellow	**6**
6	Metasoma yellow without a brown patch; MV 1.7× SMV (Zhu and Huang 2003: 447, fig. 17)	***D.platys* Zhu & Huang**
–	Metasoma yellow with a brown median patch; MV at most 1.2× SMV (e.g., Figs 12, 14, 15)	**7**
7	Antenna with scape, pedicel and F1 yellow, F2–F6 brown (Zhu and Huang 2003: 447, fig. 8); POL 2.8× OOL	***D.aequus* Zhu & Huang**
–	Antenna with scape yellow, pedicel and flagellum yellowish-brown (Fig. 13); POL 2.4× OOL	***D.hainana* sp. nov.**

### ﻿ Diglyphomorphomyia depressa sp. nov.

Taxon classificationAnimaliaHymenopteraEulophidae

D4266C38-A900-5F9A-91CD-EB46B12CAFAF

http://zoobank.org/DEFBC4FD-0A88-494A-B029-34E61DD671EC

Figs 1–4

#### Type material.

***Holotype***, ♀ [NEFU; on card], China, Sichuan Province, Guangyuan City, Qingchuan County, 21 VIII 2015, leg. Ye Chen and Chao Zhang, by sweeping. ***Paratypes***: 2♀ [on slides], China, Liaoning Province, Anshan City, Tiedong District, Gudaoguan, 19–21 VI 2015, leg. Yan Gao and Hui Geng, by yellow pan trapping.

#### Diagnosis.

Head dark brown. Antennae yellowish except F3, F4 brownish, club brownish except yellowish apex. Mesosoma yellow except propodeum brown. Midlobe of mesoscutum with two pairs of long setae. Scutellum with a longitudinal depression in anterior 2/3.

#### Description.

**Female.** Body length 2.4 mm, fore wing length 1.7 mm. Head dark brown. Antennae yellowish with F3 and F4 brownish, club brownish except yellowish apex. Mesoscutum, axillae and scutellum yellow. Propodeum brown. Legs yellowish. Gaster mostly yellow with two brown transverse bands posteriorly and margins brown. Ovipositor black.

**Figures 1–4.**
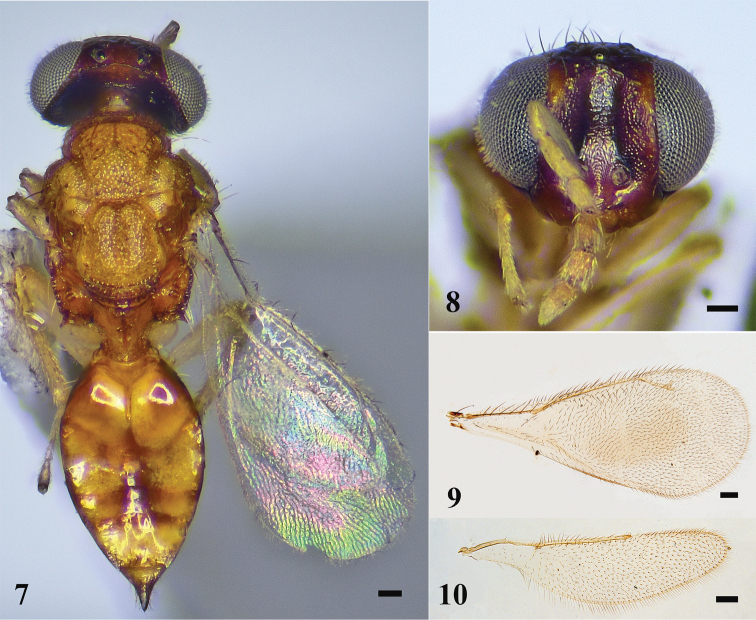
*D.depressa* sp. nov., female, holotype **1** habitus in dorsal view **2** head in frontal view **3** fore wing **4** hind wing. Scale bars: 100 μm.

***Head*** (Fig. 2) 1.2× as wide as high in frontal view and ~ 2.2× as wide as long in dorsal view. Lower face smooth, frons weakly reticulate. Vertex with engraved reticulation, meshes isodiametric, and scattered setae. POL 1.8× OOL. Malar space 0.4× eye height. Occiput weakly reticulate. Relative measurements (length: width): scape = 28: 5; pedicel = 10: 5; F1 = 16: 5; F2 = 11: 5; F3 = 12: 7; F4 = 10: 7; clava = 16: 7.

***Mesosoma*** (Fig. 1). Midlobe of mesoscutum punctate with two pairs of long setae, lateral lobe of mesoscutum reticulate. Notauli ending laterad to inner angles of axilla. Axillae weakly reticulate and separated from each other. Scutellum with a longitudinal depression in anterior 2/3 and two pairs of long setae, sublateral grooves meet posteriorly. Spiracle with a transverse carina anteriorly, separated from metanotum by a distance shorter than a diameter of spiracle; each propodeal callus with seven long setae.

***Wings*.** Fore wing (Fig. 3) 2.3× as long as wide. Speculum narrow. Relative measurements (length): SMV = 43; MV = 40; PMV = 22; STV = 16. Hind wing (Fig. 4) ~ 4.0× as long as wide.

***Metasoma*** (Fig. 1). Gaster ovate, 1.8× as long as wide, and 1.1× as long as mesosoma. Ovipositor exserted beyond apex of gaster.

**Male.** Unknown.

#### Host.

Unknown.

#### Distribution.

China (Sichuan, Liaoning).

#### Etymology.

Latin: *depressum* = depression, sink; and refers to the longitudinal depression in anterior 2/3 of scutellum.

#### Remarks.

This new species differs from all other known members of the genus in having a scutellum with a longitudinal depression on its anterior 2/3.

### ﻿ Diglyphomorphomyia fossa sp. nov.

Taxon classificationAnimaliaHymenopteraEulophidae

D444F917-6CBF-5496-9A1F-17D9C1A099A9

http://zoobank.org/808A2C5F-15CC-4BB4-BB58-1237002F6954

Figs 5–10

#### Type material.

***Holotype***, ♀ [NEFU; on card], China, Shandong Province, Qingdao City, Huangdao District, Dazhu Mountain, 22–24 V 2014, leg. Guo-Hao Zu, Xiang-Xiang Jing and Si-Zhu Liu, by yellow pan trapping. ***Paratypes***: 1♀ [on slide], same data as holotype; 1♀ [on slide], CHINA, Shandong Province, Qingdao City, Huangdao District, Xiaozhu Mountain, 18–20 V 2014, leg. Guo-Hao Zu, Xiang-Xiang Jing and Si-Zhu Liu, by yellow pan trapping; 4♀ [on card], China, Shandong Province, Qingdao City, Laoshan District, Beijiushui, 1–3 VIII 2014, leg. Guo-Hao Zu and Ye Chen, by yellow pan trapping; 1♀ [on card], China, Shandong Province, Qingdao City, Laoshan District, Dahedong, 8–10 VII 2014, leg. Chao Zhang, Si-Zhu Liu and Ye Chen, by yellow pan trapping.

**Figures 5–10.**
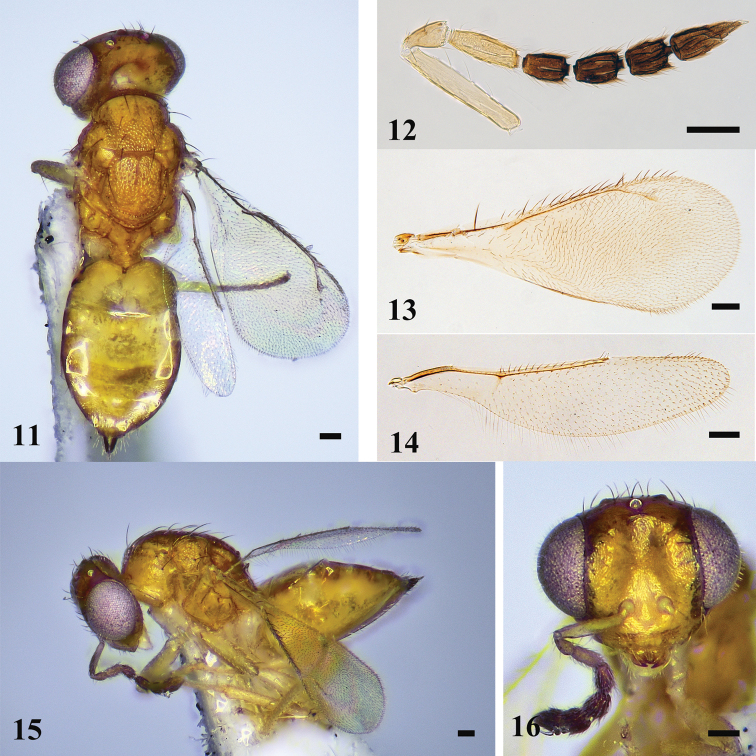
*D.fossa* sp. nov., female, holotype **5** habitus in dorsal view **6** antenna **7** fore wing **8** hind wing **9** habitus in lateral view **10** head in frontal view. Scale bars: 100 μm.

#### Diagnosis.

Antenna with scape, pedicel and F1 yellow and rest of flagellomeres dark brown. Scutellum with median groove in approximately anterior 1/2. Legs mostly yellowish with fore and hind coxae yellowish white. Gaster yellowish except margins brown.

#### Description.

**Female.** Body length 1.9 mm, fore wing length 1.3 mm. Body yellow. Mandibles yellow with teeth brown. Antenna with scape, pedicel, and F1 yellow and remainder of flagellomeres dark brown. Legs mostly yellowish except fore and hind coxae yellowish white. Gaster yellowish except margins brown. Ovipositor black.

***Head*** (Fig. 10) 1.3× as wide as high in frontal view and ~ 2.3× as wide as long in dorsal view. Lower face and vertex transversely reticulate, frons weakly sculptured. POL 2.4× OOL. Malar space 0.4× eye height. Occiput weakly reticulate. Relative measurements (length: width): scape = 35: 6; pedicel = 11: 6; F1 = 19: 6; F2 = 12: 7; F3 =13: 8; F4 = 12: 8; clava = 20: 8.

***Mesosoma*** (Fig. 5). Midlobe of mesoscutum punctate with three pairs of long setae, lateral lobe of mesoscutum reticulate. Notauli ending laterad to inner angles of axilla. Axillae weakly reticulate and separated from each other. Scutellum with median groove in approximately anterior 1/2 and two pairs of long setae, sublateral grooves meet posteriorly. Spiracle with a transverse carina anteriorly, separated from metanotum by a distance almost as long as a diameter of spiracle; each propodeal callus with six setae.

***Wings*.** Fore wing (Figs 5, 7) 2.6× as long as wide. Relative measurements (length): SMV = 33; MV= 45; PMV= 17; STV= 12. Hind wing (Figs 5, 8) ~ 4.5× as long as wide.

***Metasoma*** (Fig. 5). Gaster ovate, 1.7× as long as wide, and 1.2× as along as mesosoma. Ovipositor exserted beyond apex of gaster.

**Male.** Unknown.

#### Host.

Unknown.

#### Distribution.

China (Shandong).

#### Etymology.

Latin: *fossa* = ditch, trench; and refers to the median groove in the approximate anterior 1/2 of scutellum.

#### Remarks.

*Diglyphomorphomyiafossa* is similar to *D.aequus* Zhu & Huang, 2003 in sharing the antenna with a yellow scape, pedicel, and F1, while the remaining segments are brown; and the notauli straight and converging posteriorly to meet laterad to inner angles of axillae, but the new species can be separated from *D.aequus* by the following combination of characters: legs yellowish with fore and hind coxae yellowish white (legs yellow in *D.aequus*); gaster yellowish except margins brown (gaster yellow with a brown patch in *D.aequus*); gaster 1.7× as long as wide (gaster 1.4× as long as wide in *D.aequus*).

### ﻿ Diglyphomorphomyia hainana sp. nov.

Taxon classificationAnimaliaHymenopteraEulophidae

A4C51D5C-10FD-5A12-9A47-46A2B85892F0

http://zoobank.org/B3718EA6-954C-48D6-8741-A608907D1F9C

Figs 11–16

#### Type material.

***Holotype***, ♀ [NEFU; on card], China, Hainan Province, Wenchang City, Dongjiao Town, 22–24 IV 2019, leg. Yu-Ting Jiang, by yellow pan trapping. ***Paratypes***: 2♀ [on slides], same data as holotype.

**Figures 11–16.**
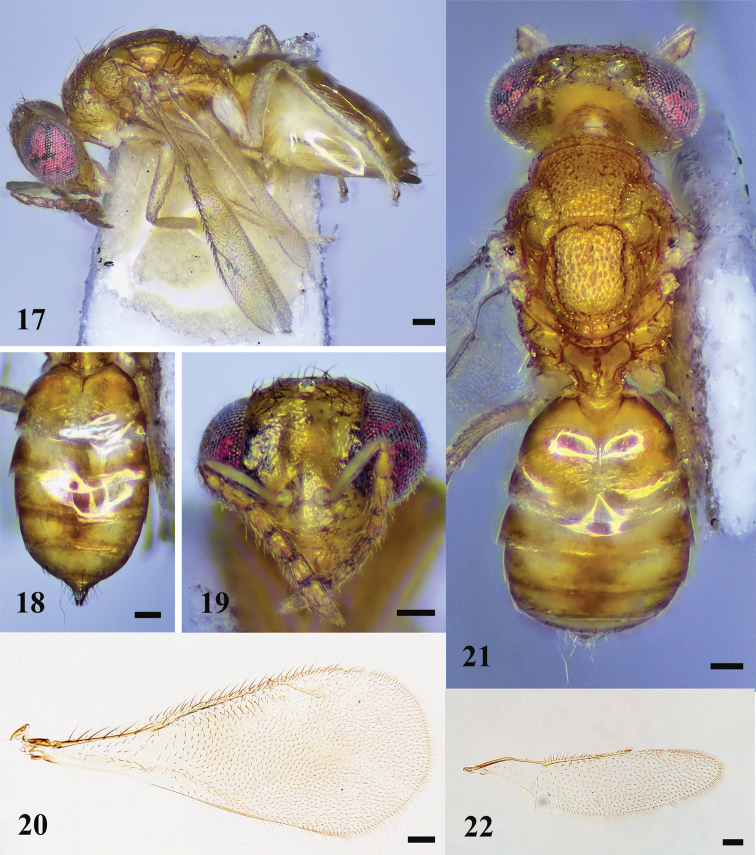
*D.hainana* sp. nov., female, holotype **11** habitus in lateral view **12** gaster in dorsal view **13** head in frontal view **14** fore wing **15** habitus in dorsal view **16** hind wing. Scale bars: 100 μm.

#### Diagnosis.

Antennae yellowish brown with scape yellow. Midlobe of mesoscutum with two pairs of long setae. Legs mostly yellowish white with fore and hind coxae white, mid coxae yellow.

#### Description.

**Female.** Body length 1.8 mm, fore wing length 1.5 mm. Body yellow. Antennae yellowish brown with scape yellow. Legs mostly yellowish white except fore and hind coxae white, mid coxae yellow. Gaster dorsally mostly yellow with a brown patch and lateral margins brown, ventrally mostly whitish.

***Head*** (Figs 11, 13) 1.4× as wide as high in frontal view and 2.4× as wide as long in dorsal view. Frons weakly sculptured. Vertex with engraved reticulation, meshes isodiametric. POL 2.4× OOL. Malar space 0.3× eye height. Occiput weakly reticulate. Relative measurements (length: width): scape = 29: 5; pedicel = 11: 5; F1 = 13: 5; F2 = 10: 5; F3 = 10: 6; F4 = 9: 6; clava = 15: 5.

***Mesosoma*** (Fig. 15). Notauli straight, ending laterad to inner angle of axilla. Midlobe of mesoscutum punctate with two pairs of long setae, lateral lobe of mesoscutum reticulate. Axillae weakly reticulate and separated from each other. Scutellum longer than mesoscutum with two pairs of long setae, sublateral grooves meet posteriorly. Spiracle with a transverse carina anteriorly, slightly separated from metanotum by a distance shorter than a diameter of spiracle; each propodeal callus with seven or eight setae.

***Wings*.** Fore wing (Fig. 14) 2.3× as long as wide. Speculum narrow. Relative measurements (length): SMV = 35; MV= 36; PMV= 16; STV= 12. Hind wing (Fig. 16) ~ 4.0× as long as wide.

***Metasoma*** (Fig. 12). Gaster ovate; 1.8× as long as wide and 1.3× as long as mesosoma. Ovipositor exserted beyond apex of gaster.

**Male.** Unknown.

#### Host.

Unknown.

#### Distribution.

China (Hainan).

#### Etymology.

The specific name is derived from the type locality.

#### Remarks.

*Diglyphomorphomyiahainana* is similar to *D.aequus* Zhu & Huang, 2003 in sharing a body that is mostly yellow, and a gaster bearing a brown patch, but can be separated from *D.aequus* by the following combination of characters: antennae yellowish brown with scape yellow (antenna brown with scape, pedicel, and F1 yellow in *D.aequus*); POL 2.4× OOL (POL 2.8× OOL in *D.aequus*); legs mostly yellowish white with fore and hind coxae white, mid coxae yellow (legs yellow in *D.aequus*).

### ﻿ Diglyphomorphomyia octoseta sp. nov.

Taxon classificationAnimaliaHymenopteraEulophidae

B3CA7D64-8A39-5BA2-BEE4-B3F04BF5D28D

http://zoobank.org/66B78964-DF2B-469D-AA6E-FDC23D26B523

Figs 17–22

#### Type material.

***Holotype***, ♀ [NEFU; on card], China, Jiangxi Province, Yichun City, Guanshan National Nature Reserve, 29 VIII 2017, leg. Guang-Xin Wang and Wen-Jian Li, by yellow pan trapping. ***Paratype***: 1♀ [on slide], same data as holotype.

**Figures 17–22.**
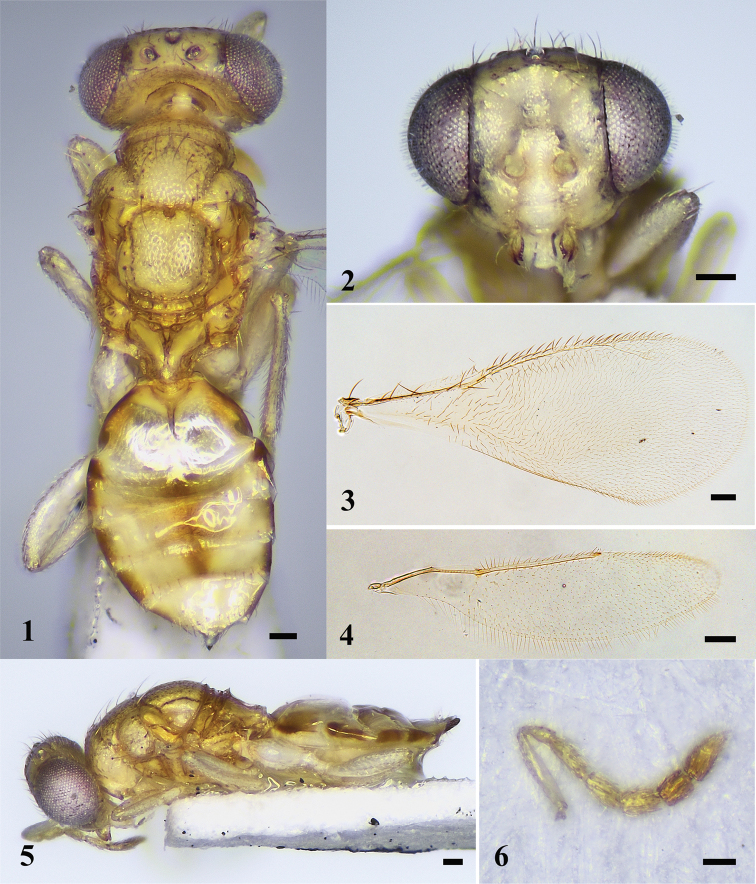
*D.octoseta* sp. nov., female, holotype **17** habitus in dorsal view **18** head in frontal view **19** fore wing **20** hind wing **21** habitus in lateral view **22** antenna. Scale bars: 100 μm.

#### Diagnosis.

The new species is easily distinguished from the other known members of the genus by the following combination of characters: Antenna with scape white, pedicel, F1–F3 pale yellow, clava brown except apex yellowish. Midlobe of mesoscutum punctate with four pairs of long setae. Propodeum with median carina bifurcate anteriorly and meeting a perpendicular lamina, anteromedially with two dorsal pits. Legs mostly white.

#### Description.

**Female.** Body length 2.2 mm, fore wing length 1.6 mm. Body pale yellow. Head yellowish-white. Antenna with scape white, pedicel, F1–F3 pale yellow, F4 and clava brown except apex yellowish. Mandibles yellow with teeth brown. Legs mostly white. Gaster mostly yellowish white, with a median stripe and lateral margins brown.

***Head*** (Fig. 18) 1.4× as wide as high in frontal view and 2.3× as wide as long in dorsal view. Frons smooth to alutaceous with a row of setae along eye margin. Vertex smooth. POL 1.8× OOL. Malar space 0.4× eye height. Occiput weakly reticulate. Relative measurements (length: width): scape = 28: 5; pedicel = 9: 5; F1 = 14: 5; F2 = 11: 6; F3 = 11: 7; F4 = 11: 7; clava = 19: 7.

***Mesosoma*** (Fig. 17). Midlobe of mesoscutum punctate with 4 pairs of long setae, lateral lobe of mesoscutum reticulate. Notauli ending laterad to inner angles of axilla. Axillae weakly reticulated and separated from each other. Scutellum longer than mesoscutum with 2 pairs of long setae, sublateral grooves meet posteriorly. Propodeum smooth, with a median carina bifurcating anteriorly and meeting a perpendicular lamina, anteromedially with two dorsal pits; plicae distinct with a transverse carina joining outer margins; another transverse carina present anterior to spiracle; separated from metanotum by a distance as long as a diameter of spiracle; each propodeal callus with seven long setae.

***Wings*.** Fore wing (Fig. 19) 2.4× as long as wide. Relative measurements (length): SMV = 63; MV = 57; PMV = 25; STV = 22. Hind wing (Fig. 20) 3.9× as long as wide.

***Metasoma*** (Fig. 17). Gaster ovate, 1.5× as long as wide and as long as mesosoma. Ovipositor exserted beyond apex of gaster.

**Male.** Unknown.

#### Host.

Unknown.

#### Distribution.

China (Jiangxi).

#### Etymology.

Latin: *octo* = eight; and refers to the midlobe of mesoscutum with four pairs of long setae.

#### Remarks.

*Diglyphomorphomyiaoctoseta* is similar to *D.kairali* Narendran & Girish Kumar in sharing a propodeum with a median carina bifurcating anteriorly and meeting a perpendicular lamina, but can be separated from the latter by the following combination of characters: fore wing hyaline without infuscation (fore wing with brown infuscation a short distance below STV in *D.kairali*); antenna with scape white, pedicel and F1–F3 pale yellow, clava brown except apex yellowish (antenna brownish black with scape and pedicel pale brownish yellow in *D.kairali*); midlobe of mesoscutum with four pairs of long setae (three pairs of long setae in *D.kairali*).

## Supplementary Material

XML Treatment for Diglyphomorphomyia

XML Treatment for Diglyphomorphomyia depressa

XML Treatment for Diglyphomorphomyia fossa

XML Treatment for Diglyphomorphomyia hainana

XML Treatment for Diglyphomorphomyia octoseta
